# Disentangling the role of sedation from improving sleep quality in crisis care

**DOI:** 10.1192/bjb.2025.32

**Published:** 2025-06

**Authors:** Jacob D. King

**Affiliations:** Division of Psychiatry, Imperial College London, London, UK

## Response to: Use of Promethazine from the perspective of resident psychiatry doctors (Corti et al)

It has been good to hear the positive response to this Against the Stream article, for what is, as far as I can tell, a distinctively British phenomenon.

These authors raise an important point about the suitability of psychological and behaviour interventions for people in psychiatric crisis. Indeed most CBT-insomnia programmes for people using psychiatric services suggest that if a person has had a period of crisis care within the preceding few weeks they are unlikely to be able to commit as meaningfully as possible to the kinds of behaviour changes required in a full CBTi programme. Having said this I disagree that CBTi is categorically not feasible on inpatient wards or under the care of community crisis services. While the evidence is limited, there are a number of good examples of delivering CBTi as part of psychiatric inpatient care.^1^ It is generally felt that outside of specialist chromotherapeutic scenarios sleep restriction techniques for people who are psychiatric inpatients could be actively unhelpful. But core psychoeducation components to bust the commonly held myths about sleep, as well as some manageable strategies with stimulus control are likely to be possible and well tolerated by some patients in crisis. Moreover responding to the often multifaceted nature of sleep problems, psychological interventions for sleep have been considered as modular,^2^ and personalised optional ‘modules’ focus on, for example, nightmares, or circadian dysfunction, some of which are likely to be helpful.

It is worth remembering that the treatment of sleeping difficulties among patients on inpatient wards has been the focus of much literature and recommendations exist,^3^ particularly environmental adjustments to ensure that people are undisturbed unless necessary, and that they feel relaxed and safe.^4^ But perhaps most importantly, as psychiatrists our job is in the assessment and treatment of mental illness, which in itself is correspondingly likely to improve the sleep quality for some, but not all, our patients.^5^

I agree with these authors that sedation is indeed likely to be more helpful than little to no sleep in many acute situations, but there is a risk-benefit. As part of a broader discussion of the role of sedative agents and sleep in psychiatric crisis, it would be helpful if further discussion and research clarified more specific scenarios in which promethazine use might be indicated. But before this, clinical trials of promethazine in sleep outcomes and in crisis management, as well as its effect of sleep architecture and the long-term effects of use ought be established before we can really be informed as to the risk-benefit.
